# A randomised controlled trial of the effect of intra-articular lidocaine on pain scores in inflammatory arthritis

**DOI:** 10.1097/j.pain.0000000000003291

**Published:** 2024-06-17

**Authors:** Zoe Rutter-Locher, Sam Norton, Franziska Denk, Stephen McMahon, Leonie S. Taams, Bruce W. Kirkham, Kirsty Bannister

**Affiliations:** a Rheumatology Department, Guy's and St Thomas' NHS Trust, London, United Kingdom; b Department Inflammation Biology, School of Immunology and Microbial Sciences, Faculty of Life Sciences and Medicine, King's College University, London, United Kingdom; c Centre for Rheumatic Diseases, King's College London, London, United Kingdom; d Wolfson Centre for Age-Related Diseases, Guy's Campus, King's College London, London, United Kingdom

**Keywords:** Intra-articular lidocaine, Rheumatoid arthritis, Inflammatory arthritis, Central pain mechanisms

## Abstract

Chronic pain in inflammatory arthritis (IA) reflects a complex interplay between active disease in a peripheral joint and central pronociceptive mechanisms. Because intra-articular lidocaine may be used to abolish joint-specific peripheral input to the central nervous system, we aimed to validate its use as a clinical tool to identify those patients with IA whose pain likely incorporates centrally mediated mechanisms. We began by investigating whether there was a placebo response of intra-articular injection in patients with IA 1:1 randomised to receive intra-articular lidocaine or control (0.9% saline). After, in a larger patient cohort not randomized to placebo vs lidocaine groups, we tested whether patients with IA could be stratified into 2 cohorts based on their response to intra-articular lidocaine according to markers of centrally mediated pain. To this end, we evaluated postlidocaine pain numerical rating scale (NRS) scores alongside baseline painDETECT, fibromyalgia criteria fulfillment, and quantitative sensory testing outcomes. Numerical rating scale scores were collected at baseline and 3-, 5-, and 10-minutes postinjection. Firstly, the placebo effect of intra-articular injection was low: compared to baseline, the mean pain NRS score 5-minutes postinjection was reduced by 3.5 points in the lidocaine group vs 1.2 points in the control group. Secondly, postlidocaine NRS scores were significantly higher in those with a high (>18) baseline painDETECT score, fibromyalgia, and low-pressure pain threshold at the trapezius (*P* = 0.002, *P* = 0.001, *P* = 0.005, respectively). Persistent high pain after intra-articular lidocaine injection could be used as an indicator of pronociceptive mechanisms that are centrally mediated, informing centrally targeted analgesic strategies.

## Abstract 2

Supplemental Digital Content is Available in the Text.

Patients with inflammatory arthritis who have central pain markers show persistent high pain post intra-articular lidocaine, helping to identify the contribution of central vs peripheral processes.

## 1. Introduction

Chronic pain in inflammatory arthritis (IA) arises because of a complex interplay between active disease in peripheral joints and central pain processing mechanisms.^11^ This interplay differs between patients, meaning that identifying the predominant underlying pain-driving mechanism, while difficult, is crucial if optimal pharmacotherapies are to be prescribed. Currently, peripherally mediated pain in IA is inferred indirectly by examining the degree of inflammation or joint damage (as assessed by ultrasound).^6^ In contrast, although validated questionnaires and quantitative sensory testing (QST) can be used to determine the trait vs state nature of the pain experience and the integrity of pain processing circuits, respectively, there is no “gold standard” method to infer the presence of centrally mediated pain in IA despite its predicted prevalence in up to 40% of patients with IA.^17^ Pinpointing peripheral vs central nervous system contributions to the pain state could aid phenotyping of pain, where stratifying patients into appropriate mechanism-based pain “cohorts” is a key goal and could aid targeted analgesic treatment for the individual.^23,26^

Lidocaine blocks voltage-gated sodium channels leading to a reversible blockade of action potential propagation in peripheral nerves at the site of injection.^18,25^ Coupled with its short-lasting action, administration of intra-articular lidocaine in a diseased joint should largely abolish the peripheral drive from that joint and, theoretically, lead us towards the possibility of uncoupling centrally vs peripherally mediated pain. Lidocaine administration was previously validated to identify peripherally mediated pain in syndromes including peripheral neuropathic pain, phantom limb pain, and bladder pain syndrome,^9,13,21^ and we demonstrated that patients with bladder pain syndrome could be categorised as having predominantly peripherally vs centrally mediated pain after intravesical lidocaine infiltration.^13^ Interestingly, those patients who did not respond to lidocaine (ie, pain scores were not reduced by >50%) were 2.7 times more likely to experience other central sensitivity syndromes, indicative of centrally mediated pain.^13^

In the present study, we hypothesised that a population of patients with IA could be stratified into 2 cohorts based on their response to intra-articular lidocaine, postulating that pain numerical rating scale (NRS) scores would reduce in all patients due to lidocaine blocking a large component of the peripherally mediated pain, but that those with contributing centrally mediated pain would report higher ongoing postinjection pain in a manner linked with markers of centrally mediated pain processing. We began by performing a placebo response study investigating patient responses to intra-articular injection of lidocaine vs a control injection of saline. Next, we performed a second analysis in a larger independent patient cohort enrolled in the “Pain Phenotypes and their Underlying Mechanisms in Inflammatory Arthritis” (PUMIA) study, where the study protocol did not incorporate randomization to placebo vs lidocaine groups, and we were able to assess postlidocaine injection NRS scores alongside painDETECT (a validated questionnaire used to assess the presence of possible neuropathic like pain, as a proxy marker of centrally mediated pain^15^) scores, fulfilment of fibromyalgia criteria and dynamic quantitative sensory testing outcomes. Broadly, we hypothesised that those patients with a high (>18) baseline painDETECT score, where additional markers of centrally mediated pain were also indicated, would, postlidocaine injection, rate their pain higher than those patients with a low (≤18) baseline painDETECT score.

## 2. Methods

### 2.1. Placebo Response Study

#### 2.1.1. Trial design

This two-armed parallel group randomised controlled trial was approved by Yorkshire and The Humber- Sheffield research ethics committee (REC reference 22/YH/0051). All patients gave written informed consent. This study was preregistered on www.clinicaltrials.gov prior to first patient enrollment (Identifier NCT05302232, Unique protocol ID 311,106) and has been designed and reported in line with the CONSORT 2010 guidelines and checklist.^7,12^

#### 2.1.2. Participants

Patients with a diagnosis of IA, including but not limited to rheumatoid arthritis and psoriatic arthritis, with a numerical rating scale (NRS) pain score >3/10 and who required an intra-articular steroid injection as recommended by the direct care team were recruited from Guy's Hospital Rheumatology department. We excluded those with underlying joint damage identified on routine x-ray, those under 18 years of age, and those requiring shoulder or proximal interphalangeal joint injection.

#### 2.1.3. Randomization and interventions

Patients were block randomized in a 1:1 allocation ratio, using the online randomization service sealed envelope (Sealedenvelope.com), to receive either intra-articular 1% lidocaine plus steroid or, as a placebo control, 0.9% saline plus steroid. Patients only were blinded to study group. For pragmatic reasons, the outcome assessor also administered the injections and was thus unblinded. Standardised amounts of steroid and lidocaine were administered for each joint (see Table 1, Supplemental Digital Content, http://links.lww.com/PAIN/C70).

#### 2.1.4. Outcomes

Demographics including age, sex, and diagnosis were collected. Pain scores (NRS 0-10) at rest in the chosen joint were collected prior to injection and at 3, 5, and 10 minutes postinjection, when the lidocaine would be expected to have an analgesic effect.^4^ Because the steroid is slower acting than lidocaine, it should not have a beneficial effect on pain scores within 10 minutes.^18,25^ Improvement in pain rating within 10 minutes by patients who received steroid only would, therefore, be because of placebo effect and random variation alone. Needle placement within the joint was confirmed by fluid aspiration.

Participants in the placebo study also completed the painDETECT questionnaire at baseline. This questionnaire assesses possible neuropathic like pain as a proxy for centrally mediated pain and has sensitivity and specificity of 84%, using clinician-assessed diagnosis of neuropathic pain as the gold standard.^8^ For the purposes of this study, painDETECT scores were grouped into high (>18) or low (≤18) likelihood of neuropathic like pain.

#### 2.1.5. Sample size

Using our pilot data, we estimated that 80 patients would be required (40 in each group) to achieve 80% power to detect a difference in NRS of at least 1.5 points between the experimental group (lidocaine) compared to the control group at the 5% alpha level. This was based on the expectation that we would perform an ANCOVA and on assuming a SD of 2.9 for the NRS, and a pre–post-NRS correlation of r = 0.4. A planned interim analysis was conducted at a total sample size of 50 (ie, 25 per group). To account for this analysis, the critical alpha level was set at *P* = 0.03 based on the Pocock method for alpha control (ie, multiple testing) in sequential analyses. The interim analysis met the stopping criteria and thus recruitment was halted early, and the final sample size was 51.

#### 2.1.6. Statistical methods

Means with standard deviation (SD) are used to describe continuous demographics and painDETECT scores. A linear mixed effects model was used to estimate between group differences (lidocaine vs control) at each of the postintervention time points, adjusted for the baseline level of the outcome. Specifically, group allocation and assessment time were included as categorical variables using dummy coding along with a time-by-group interaction terms. Baseline NRS was included as a covariate. A random intercept was included to account for the repeated assessments within individuals. Further analysis extended the model by including a dummy coded covariate for high vs low painDETECT score and three-way interaction terms between painDETECT with group and time. This allows for the interrogation of lidocaine effect heterogeneity by estimating specific lidocaine treatment effects for those with high vs low painDETECT scores at each time point. Analysis was performed in Stata V 17.1. For the between group difference (lidocaine vs control), the significance threshold was set at *P* < 0.03 because of the sequential design used and *P* < 0.05 for all other analyses.

### 2.2. Pain phenotypes and their underlying mechanisms in inflammatory arthritis study

#### 2.2.1. Trial design

Supplementary analysis was performed using data from 40 patients enrolled in the “Pain phenotypes and their Underlying Mechanisms in Inflammatory Arthritis” study (PUMIA), a single site observational cohort study of patients with inflammatory arthritis. Pain phenotypes and their Underlying Mechanisms in Inflammatory Arthritis study received approval from Bromley research ethics committee and the Health Research Authority (REC 21/LO/0712). All patients gave written informed consent.

#### 2.2.2. Participants

Patients with a diagnosis of IA with a numerical rating scale (NRS) pain score >3/10 and who required an intra-articular steroid injection as recommended by their direct care team were recruited. Exclusion criteria mirrored those in the RCT but additionally excluded those who were receiving noninflammatory arthritis–related immunosuppressant therapy, undergoing current or recent (last 90 days) treatment with investigational agents, using opioids, gabapentin, or pregabalin within 24 hours prior to assessment or having a history of severe peripheral vascular disease or peripheral neuropathy.

#### 2.2.3. Intervention

This was not a randomized study. All patients received intra-articular 1% lidocaine along with steroid injection, using the same technique and standardized amounts as the placebo response study.

#### 2.2.4. Outcomes

As in the placebo response study, pain scores (NRS 0-10) at rest in the chosen joint were collected prior to injection and at 3-, 5- and 10-minutes postinjection. In addition to collection of the painDETECT questionnaire, fibromyalgia status was determined according to the 2016 revision of the ACR 2010 modified fibromyalgia diagnostic criteria^24^ and QST was performed. Quantitative sensory testing included evaluation of pressure pain thresholds at a nonarticular site as a marker of widespread pain sensitisation (the bilateral trapezius, taken as an average), temporal summation of pain (TSP), and conditioned pain modulation (CPM) using cuff algometry, as detailed in previous publications^20^

#### 2.2.5. Sample size

Given the nature of the supplementary analysis, no a priori power calculation was performed. The sample size provides 73% power to detect a correlation of at least 0.4 based at the 5% (two-sided) significance level.

#### 2.2.6. Statistical methods

A linear mixed effects model estimated between group differences in NRS by painDETECT group (high vs low) at 3-, 5- and 10-minutes postlidocaine injection. Specifically, painDETECT group and time were included as dummy coded variables along with interaction terms. The analyses were repeated replacing painDETECT group with other related pain variables: positive or negative for fulfillment of fibromyalgia criteria, pressure pain threshold (PPT) high vs low group (determined as above or below median PPT at the trapezius in the absence of normative data), positive or negative for facilitated TSP (determined using ratio >2.48 to represent facilitated TSP in the absence of normative data^20^), and responder or nonresponder to CPM (determined using CPM response >20% of the baseline pressure tolerance threshold to represent responders and CPM response <20% of the baseline pressure tolerance threshold to represent nonresponders in the absence of normative data^20^). Using these additional groupings, positive fulfillment of fibromyalgia criteria, PPT low group, positive for facilitated TSP, and nonresponder to CPM were deemed to be proxy measures of centrally mediated pain.

## 3. Results

### 3.1. Placebo response study

#### 3.1.1. Recruitment and participant flow

Fifty-one patients were recruited between April and October 2022, 26 in the treatment (lidocaine) group and 25 in the control group. No participants were lost or excluded postrandomisation as randomisation and intra-articular injection occurred on the same study visit.

#### 3.1.2. Baseline data

Demographics are shown in Table 1. A total of 64.7% were female and mean age was 53.4 years with a large range of 26 to 84 years, in keeping with the disease demographics. Most participants had rheumatoid arthritis (61%) or psoriatic arthritis (19.6%). The most common joints injected were the knee (57%) or wrist (28%). Preinjection NRS scores were high, as would be expected for patients requiring joint injection.

**Table 1:** Demographics of placebo response study population.

Variable	Lidocaine (n = 26)	Control (n = 25)	Total (n = 51)
Age, y (mean, SD)	51 (15)	54 (17)	53.4 (15)
Sex, F (N, %)	17 (65%)	16 (64%)	33 (64.7%)
Diagnosis, (N, %)			
EIA	1 (4%)	1 (4%)	2 (3.9%)
Seropositive RA	10 (38%)	8 (32%)	18 (35.2%)
Seronegative RA	7 (27%)	6 (24%)	13 (25.4%)
PsA	3 (12%)	7 (28%)	10 (19.6%)
Peripheral SpA	4 (15%)	1 (4%)	5 (9.8%)
JIA	1 (4%)	1 (4%)	2 (3.9%)
IBD-related arthritis	0 (0%)	1 (4%)	1 (2.0%)
Preinjection pain NRS score, mean (SD)	7.2 (2.0)	6.4 (2.5)	6.9 (2.3)
Joint			
Knee	14 (54%)	15 (60%)	29 (57%)
Wrist	7 (27%)	7 (28%)	14 (28%)
Elbow	1 (4%)	3 (12%)	4 (8%)
Ankle	3 (12%)	0 (0%)	3 (6%)
MCP	1 (4%)	0 (0%)	1 (2%)
Fluid off (mLs), mean (SD)	7.2 (13)	8.5 (20)	7.8 (16)
PainDETECT score, mean (SD)	16 (9.4)	13 (8.0)	14.7 (8.7)

Means presented with SD. There was no significant difference between variables presented in lidocaine and control group.

#### 3.1.3. Placebo effect of intra-articular injection is low

**Figure 1.**
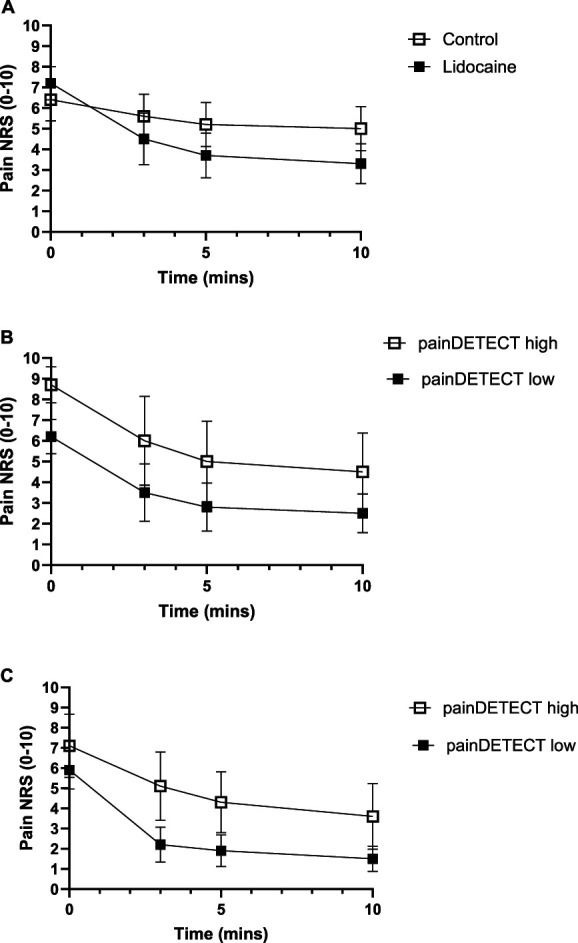
Numerical rating scale (NRS) response at 3, 5, and 10 minutes post- to intra-articular injection. (A) Pain NRS score pre- and postlidocaine vs steroid-only control injection in RCT group (mean ± 95% CI). (B) Pain NRS score pre- and postlidocaine, grouped by painDETECT low/high in first patient cohort (RCT group) (mean ± 95% CI). (C) Pain NRS score pre- and postlidocaine, grouped by painDETECT low/high in second validation cohort (PUMIA group).

The mean pain NRS score was 3.5 points lower than baseline at 5 minutes postinjection in the intra-articular lidocaine group and 1.2 points lower than baseline at 5 minutes postinjection in the steroid only placebo control group (Fig. 1A). Decomposition of the total change from baseline indicated that a 2.8 point difference (81% of the total effect) was because of the treatment effect of lidocaine and the remaining 0.7 points (19%, representing an NRS scale reduction of 1.2 points compared to baseline) placebo effect. This was the primary registered outcome.

Linear mixed effect regression, adjusting for baseline pain NRS scores, indicated that the lidocaine group had significantly lower NRS scores on average across the postinjection time points, compared to the control group (main effect *P* = 0.002). The adjusted mean differences were observed to be significant at each of the 3 assessment points with an increasing effect over time (Table 2).

**Table 2:** Adjusted mean differences for lidocaine compared to control group at different time points (raw and standardized values).

Time, mins	Raw marginal effect	Standardized marginal effect	Significance
3	1.79	0.65	*P* = 0.002
5	2.14	0.78	*P* < 0.001
10	2.40	0.87	*P* < 0.001

#### 3.1.4. Patients with a high painDETECT score report higher ongoing pain after intra-articular lidocaine

Regardless of high (>18) or low (≤18) preinjection painDETECT scores, a comparable reduction in pain NRS scores were reported by patients postlidocaine injection (Fig. 1B, 4.2 vs 3.7 points, respectively), and postplacebo injection (see supplemental digital content, Figs. 1, http://links.lww.com/PAIN/C70, 1.1 vs 1.4 points respectively). This potentially indicates a similar level of peripherally driven nociceptive pain (presumably generated by joint inflammation) in all patients. The mean NRS score 5 minutes post intra-articular lidocaine was 5 in the high painDETECT group and 2.8 in the low painDETECT group. On average, across all 3 postinjection assessments, those in the high painDETECT group had a NRS score that was 2.2 points higher than those in the low painDETECT group (*P* = 0.025, Table 3 and see supplemental digital content, Table 3, http://links.lww.com/PAIN/C70). A sensitivity analysis was run controlling for baseline NRS scores; this reduced the average difference in NRS between the groups across all 3 postinjection assessments to nonsignificant (0.5, *P* = 0.49).

**Table 3:** Results of the linear mixed effect model.

		PainDETECT high/low RCT group (n = 26)	PainDETECT high/low (n = 40)	FM +ve/−ve (n = 40)	Trapezius PPT below/above median (n = 40)	Facilitated TSP +ve/−ve (n = 32)	CPM responder/nonresponder (n = 40)
Unadjusted for preinjection NRS score	Group average	**2.2 (0.3 to 4.2)** ***P* = 0.025**	**2.5 (0.9 to 4)** ***P* = 0.002**	**2.4 (1 to 3.8)** ***P* = 0.001**	**1.9 (0.6 to 3.3)** ***P* = 0.005**	−1.6 (−4.0 to 0.8) *P* = 0.182	−0.7 (−2 to 0.6) *P* = 0.307
Adjusted for preinjection NRS score	Group average	0.5 (−0.9 to 1.8) *P* = 0.49	**1.9 (0.8 to 3)** ***P* = 0.001**	**1.6 (0.3** to **2.9)** ***P* = 0.016**	1.2 (0.0 to 2.4) *P* = 0.052	−1.6 (−3.4 to 0.3) *P* = 0.102	−0.2 (−1.2 to 0.8) *P* = 0.693

The table presents the difference in postinjection pain scores, represented as group averages accompanied by 95% confidence intervals. Initial results are provided without adjustments for preinjection pain NRS scores, followed by data that are adjusted for these scores. The first column displays outcomes from patients in the RCT, categorized based on their painDETECT high/low status. Subsequent columns show data from the second validation cohort (PUMIA), grouped not only by painDETECT high/low but also by other indicators of centrally mediated pain.

Entries highlighted in bold indicate significance at the 5% level.

RCT, randomized controlled trial; FM, fulfillment of fibromyalgia criteria; TSP, temporal summation of pain; CPM, conditioned pain modulation.

### 3.2. Pain phenotypes and their underlying mechanisms in inflammatory arthritis study

#### 3.2.1. Recruitment and participant flow

Forty patients were included in the replication (validation) analysis using the PUMIA cohort, recruited between April 2022 and July 2023. All assessments were performed on the same study visit meaning that no patients were lost to follow-up.

#### 3.2.2. Baseline data

Demographics are shown in Table 4 and Table 4, supplemental digital content, http://links.lww.com/PAIN/C70. Seventy-three percentage were female and mean age was 52.7 years. Most participants had rheumatoid arthritis (83%). The most common joints injected were the wrist (58%) or knee (23%). Median PPT at the trapezius was 2.1 kg/cm^2^.

**Table 4:** Demographics of PUMIA study population.

Variable	Low painDETECT (n = 27)	High painDETECT (n = 13)	Total (n = 40)
Age, y (mean, SD)	50 (15)	58 (14)	52.7 (15)
Sex, F (N, %)	18 (67%)	11 (85%)	29 (73%)
Diagnosis, (N, %)			
EIA	4 (15%)	0 (0%)	4 (10%)
Rheumatoid arthritis	20 (74%)	13 (100%)	33 (83%)
PsA	2 (7%)	0 (0%)	2 (5%)
Peripheral SpA	1 (4%)	0 (0%)	1 (3%)
Preinjection pain NRS score (mean, SD)	5.9 (2.4)	7.1 (2.6)	6.3 (2.5)
Joint			
Knee	6 (22%)	3 (23%)	9 (23%)
Wrist	14 (52%)	9 (69%)	23 (58%)
Elbow	3 (11%)	0 (0%)	3 (8%)
Ankle	2 (7%)	0 (0%)	2 (5%)
MCP	2 (7%)	1 (8%)	3 (8%)
PainDETECT score (mean, SD)	11 (4.5)	25 (3.5)	15 (7.8)

Means presented with SD. Shown as demographics of those with low painDETECT and those with high painDETECT and then total population.

#### 3.2.3. Patients with a high painDETECT score, fibromyalgia, and low nonarticular pressure pain threshold report higher ongoing pain after intra-articular lidocaine, in a second patient cohort

Regardless of high (>18) or low (≤18) preinjection painDETECT scores, a comparable reduction in NRS scores was reported by patients postlidocaine injection (Fig. 1C). The mean NRS score at 5 minutes post intra-articular lidocaine was 4.3 in the high painDETECT group and 1.9 in the low painDETECT group. Based on a mixed effects model, those in the high painDETECT group had a postlidocaine injection NRS score that was 2.5 points higher than those in the low painDETECT group (Table 3, main effect *P* = 0.002), and NRS scores were significantly higher in the high painDETECT group at each postintervention time point (see Table 3, supplemental digital content, http://links.lww.com/PAIN/C70). Sensitivity analysis, controlling for preinjection NRS scores, revealed that, although less attenuated, in this second cohort, there was still a significant difference between pain scores post intra-articular lidocaine injection in those with high vs low painDETECT (Table 3).

**Figure 2.**
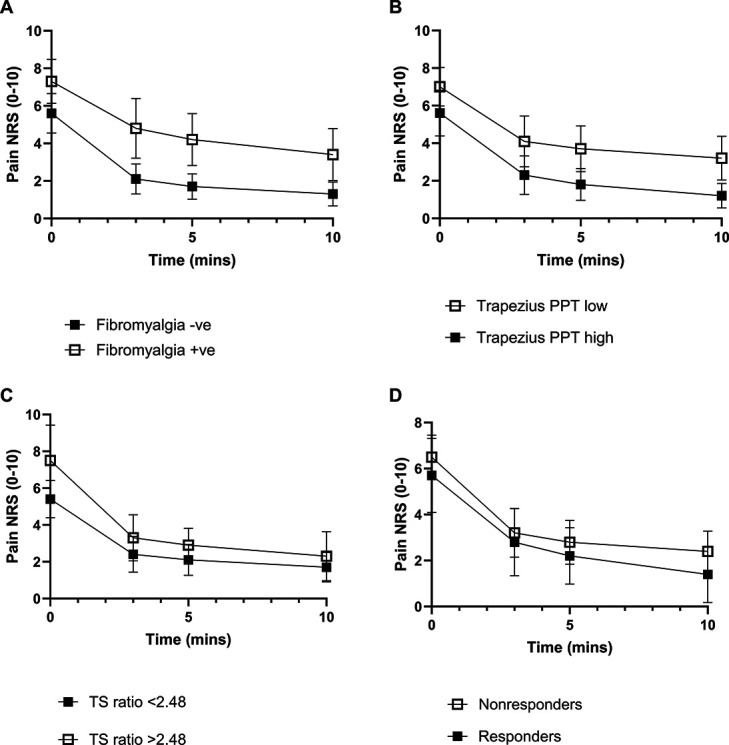
Drop in pain numerical rating scale (NRS) score pre- and postlidocaine in PUMIA patients, grouped by (A) fulfilment of fibromyalgia criteria +ve/−ve (*P* = 0.001), (B) pressure pain threshold (PPT) high vs low group (above or below median PPT at the trapezius) (*P* = 0.005), (C) fulfilment of temporal summation of pain (TS) ratio >2.48 +ve/−ve (*P* = 0.182), or (D) responder or nonresponder to conditioned pain modulation (CPM) pressure tolerance threshold (*P* = 0.307) (mean ± 95% CI).

Further analysis considered other markers of central pain processing. Average postinjection NRS scores were significantly higher in those fulfilling fibromyalgia criteria compared to those who did not, both when adjusting or not adjusting for preinjection pain scores (Fig. 2A and Table 3). Average postinjection NRS scores were also significantly higher in those with low, vs high, PPT at the trapezius, when not adjusting for preinjection NRS scores, but this effect did not survive correction for preinjection NRS scores (Fig. 2B and Table 3). Postinjection NRS scores were also numerically higher in those with facilitated TSP compared with not facilitated TSP, and those who were nonresponders to CPM compared to responders, but this was not significant (*P* = 0.182 and *P* = 0.307, respectively, Fig. 2C and 2D and Table 3). Ratings at 5 minutes post intra-articular lidocaine in those who had a high a painDETECT and who fulfilled fibromyalgia criteria consistently exceeded 4/10 (see Table 2, supplemental digital content, http://links.lww.com/PAIN/C70).

## 4. Discussion

We began our study by performing a stand-alone randomized placebo response trial to investigate responses of patients with IA to intra-articular injection of lidocaine vs a control injection of saline. We considered that the placebo response to intra-articular saline—a reduction in reported NRS (0-10) of 1.2 points—was low relative to the reduction in reported NRS of 3.5 points with lidocaine. Having demonstrated heterogeneity in lidocaine effect as evaluated by baseline painDETECT scores, next, using a protocol that incorporated assessment of centrally mediated markers of pain in a large patient cohort not randomized to placebo vs lidocaine groups, we performed a secondary analysis, which demonstrated that, postlidocaine injection, NRS scores were significantly higher in those patients with a high baseline painDETECT score, fibromyalgia, and low-pressure pain thresholds at a site distal to the joint inflammation.

The placebo response, widely studied in pain science,^10,14^ is influenced by internal factors such as patient expectation, emotions, and past experiences, as well as external factors such as verbal suggestions, social cues, and body language.^3^ It has a well-defined biological foundation that includes not only the autonomic and neuroendocrine systems but also modulatory processes involving the prefrontal cortex and the axis of the periaqueductal grey, rostroventral medulla, and spinal cord.^22^ Given this complexity, it was vital to evaluate whether any element of the pain-reducing effect of an intra-articular injection was governed by a placebo mechanism. Because, comparative to lidocaine, the placebo response to a saline intra-articular injection was low, we propose that the beneficial effect of intra-articular lidocaine on pain scores (as measured using the NRS) was mainly the result of a peripheral neurological action, rather than a distinct placebo response.

Lidocaine, as a nonselective voltage-gated sodium channel blocker, prevents depolarization and action potential propagation in all peripheral nerve fibres (including motor, sensory, and autonomic) at the site of injection.^13^ When administered into a diseased joint, therefore, it should block the majority of transmission arising from inflammation and/or joint damage driven nociception.^21^ We hypothesized that the level of pain reported postinjection might differentiate those patients with predominantly centrally, vs peripherally, mediated pain. In the absence of studies validating this use of lidocaine in IA, and having demonstrated a low placebo response of intra-articular injection, we next performed, in a large patient cohort, analysis of the pain experience before and after lidocaine injection using patient-reported questionnaires and quantitative sensory testing to study the integrity of central nervous system pain processing circuits.

PainDETECT, originally established as a validated screening tool to detect possible neuropathic pain components in patients with chronic lower back pain,^8^ emerged as a means to predict, with high sensitivity, pain type (based on symptoms) and severity. Relevant for our study, the assessed symptoms are not specific to (although more frequent in) neuropathic pain and share features of centrally mediated pain. In our study, all patients had actively inflamed joints so would be expected to have significant peripheral nociceptive pain. We observed reductions in pain scores postlidocaine injection regardless of whether the individual scored high (>18) or low (≤18) when completing the painDETECT questionnaire, possibly reflecting a similar degree of peripherally mediated pain in all patients. However, those with high painDETECT scores reported greater ongoing NRS pain scores after intra-articular lidocaine injection compared to patients with low painDETECT scores.

Central pain mechanisms are classically described as a consequence of ongoing nociceptive input^9^ (eg, after nerve injury or inflammation). Additionally, it is increasingly recognised that centrally mediated pain can result and/or be maintained independently of peripheral input. Because the analgesic effect of lidocaine is short acting, and mechanisms that underpin centrally mediated pain are not likely to reverse within the 10-minute timeframe that our postinjection pain scores were collected, we propose that people with high ongoing pain scores despite lidocaine administration are experiencing pain that is mechanistically underpinned by pronociceptive central processes not impacted by the lidocaine administration.

To address the fact that painDETECT as a screening tool in inflammatory arthritis has only limited validation against quantitative sensory testing^1^ (see the limitation section for further detail), we broadened our analysis in a second “validation” cohort including more measures indicative of maladaptive central nervous system plasticity in chronic pain states: fibromyalgia criteria, in addition to static and dynamic QST methods. Interestingly, individuals (1) stratified into the high painDETECT group (2) meeting fibromyalgia criteria and (3) displaying low nonarticular PPT that is, at the trapezius, reported higher pain NRS scores post intra-articular lidocaine. The use of static QST testing provides an inference of functionality in nervous system primary afferent fibres, where PPT measures, for example, may provide an indication of sensory gain (hyperesthesia, hyperalgesia, allodynia) or loss (hypoesthesia, hypoalgesia) of function, pointing to small and/or large diameter nerve fibre dysfunction according to a psychophysical profile. Static measures also may indicate centrally mediated pain when, as investigated in this study, thresholds deviate from normal at nondiseased sites such as the trapezius.

In contrast, in our study, differences in postinjection pain NRS levels were not statistically significant when categorized by dynamic QST measures. These included TSP and CPM, where TSP paradigm outcomes provide a proxy measure of spinal facilitatory processes, and CPM paradigm outcomes provide a proxy measure of functionality in a modulatory process that, in health, acts to inhibit spinal neuronal activity. Widespread hyperalgesia and/or the presence of centrally mediated pain was suggested in recent meta-analyses of studies of pain mechanisms in patients with IA based on questionnaire data^17^ and low PPT at extra-articular sites.^19^ However, similar to our lack of finding with regards to responses to intra-articular lidocaine when patients are grouped by TSP and CPM responses, previous studies on the roles of spinal facilitatory and descending modulatory mechanism in IA were inconclusive.^19^ Our results, which demonstrate lowered PPT at the trapezius with no indication of abnormal spinal facilitatory/brain modulatory processing, highlight that considering QST outcomes in isolation is a mistake if a mechanistic understanding of a pain-driving centrally mediated process is sought.

This study has limitations. Using our study design, it is impossible to be certain that lidocaine penetrated all nociceptors in the joint, such as those that are in the subchondral bone. However, studies to date on the effects of intra-articular lidocaine administration in OA knees suggest otherwise. Specifically, results have indicated that lidocaine does act on relevant nociceptors in the joint evidenced by both a significant reduction in pain rating according to the visual analogue scale^5^ but also higher-pressure pain thresholds at the knee and surrounding muscles.^10^ To investigate this more directly, 1 would need to adopt an alternative method of abolishing peripheral inputs, such as regional lumbar plexus blockade. Regarding our use of painDETECT (which does not include a physical examination) as a screening tool to detect possible elements of neuropathic pain in a disease state we note that, although the painDETECT questionnaire has been used in the literature to indicate central pain processes,^2,15,16^ validation against quantitative sensory testing or measures of inferred underlying mechanisms in inflammatory arthritis is limited. It is also noteworthy that we did not include here analysis of, for example, participant systemic inflammatory processes or psychological profiles. Thus, because pain, a multifactorial phenomenon, incorporates multiple somatic and nonsomatic factors, we cannot definitively conclude (in the absence of comprehensive sensory assaying) that participants who did not respond to lidocaine do not have a manifestation of pain that incorporates aspects of centrally driven pain.

## 5. Conclusions

Overall, our findings support the concept that post intra-articular lidocaine pain scores could be used to identify the contribution of central vs peripheral processes. However, no indication of the mechanism(s) underpinning the peripheral or centrally mediated pain may be gleaned from our study.

## Conflict of interest statement

The authors have no conflicts of interest to declare.

## Appendix A. Supplemental digital content

Supplemental digital content associated with this article can be found online at http://links.lww.com/PAIN/C70.

## Supplementary Material

SUPPLEMENTARY MATERIAL
